# Leptin Contributes to the Development of the Corpus Luteum

**DOI:** 10.4172/2168-9296.1000190

**Published:** 2017-12-12

**Authors:** Michelle R Garcia

**Affiliations:** Department of Animal, Rangeland and Wildlife Science, Texas A&M University-Kingsville, TX 78363, USA

**Keywords:** Leptin, Corpus luteum, Angiogenesis development

## Abstract

The mechanistic events of female infertility have been investigated for over 50 years and despite progress many causes of infertility remain elusive. However, over half of idiopathic infertility issues have been attributed to a defective ovarian tissue responsible for the maintenance of a conceptus, the corpus luteum (CL). Many CL defects are attributed, in part, to abnormal vascularization (angiogenesis), which occurs primarily during the developmental stage of the luteal lifespan. A few well-established angiogenic growth promotants have been implicated in luteal angiogenic processes but the mechanisms of the process are still under investigation. Recent evidence supports a role for the adipokine hormone leptin as a probable component in the angiogenic and developmental processes of a CL. Leptin expression is present during the developmental and maturation stages of the luteal lifespan and stimulates the expression of angiogenic hormones in the CL. Induced leptin deficient CL have a higher occurrence of abnormal, underdeveloped gross morphology and an increase in the number of large diameter vessels and large luteal cells. Leptin replacement therapy in leptin deficient CL accelerates tissue development, increasing overall tissue mass and forming a structure that resembled a mature CL during the early stages of development. Collectively, the evidence supports the supposition that leptin is involved in the angiogenic and developmental processes of luteal tissue.

## Commentary

The corpus luteum (CL) is an important ovarian tissue that secretes progesterone, a steroid hormone essential for the maintenance of pregnancy in mammals. It exhibits tumorigenic growth properties during the developmental process, doubling in size and cell number every 60-70 h [1]. In order to support the exponential tissue growth the CL is highly vascularized, having the highest rate of blood flow per unit of tissue in the female body [2]. Inappropriate vascularization leads to aberrant CL development and reduced circulating concentrations of progesterone [3]. The reduced progesterone is associated with an increased occurrence of miscarriage [4], which is not mitigated with the use of synthetic progestins in subjects suffering recurrent miscarriages [5]. Hence, understanding the underlying mechanisms of luteal development, including the angiogenic process, can potentially lead to therapies that correct luteal deficiencies and ameliorate luteal infertility. Vascularization of the CL occurs through an angiogenic process where vessels form from pre-existing vascular networks of an ovulated follicle. This process is regulated in part by the angiogenic hormones vascular endothelial growth factor (VEGF), fibroblast growth factor 2 (FGF2) and angiopoietin 1 (Ang1). Both VEGF and FGF2 promote capillary membrane destabilization, endothelial cell differentiation, proliferation, migration and vascular tube formation in human, bovine, and ovine luteal tissue [6,7]. Angiopoietin 1 then promotes the maturation and stabilization of nascent vessels through the recruitment of stromal support cells, including pericytes and smooth muscle cells [8]. Each of these angiogenic factors is regulated by the adipogenic hormone leptin, which has previously been reported to exhibit angiogenic properties in non-ovarian tissues [9,10]. The expression of leptin and its receptor have been identified in luteal tissue, but the function of leptin was believed to be limited steroidogenic regulation. However, its role in luteal steroidogeneis has proven to be moderate without the addition of growth promoting hormones [11, 12] which suggests that leptin may serve an alternate function previously overlooked that is supportive of the highly vascular tissue.

In 2014, Wiles et al. [13] reported that leptin upregulates the expression of VEGF, FGF2 and Ang1 in cultured dispersed lutea, but this stimulatory effect was limited to the early developing lutea despite sustained luteal expression of leptin and its receptor in the mature CL. This implied that leptin might be involved in luteal angiogenic processes as the CL forms. This supposition was explored by creating a leptin deficient CL with the infusion of a leptin antibody throughout the development and maturation stages of the luteal lifespan. The induced luteal leptin deficiency increased the occurrence of CL with an abnormal, persistently underdeveloped gross morphology during the late stage of the luteal lifespan, frequently resembling an early developing CL [14]. Furthermore, leptin deficiency altered the microscopic morphological landscape by increasing the number of large diameter vessels (Table 1) and population of large luteal cells (Table 1) [14]. These changes in luteal landscape may be a compensatory adaptation to the reduction in the contribution of leptin to the angiogenic processes during CL development. The adaptation may have prevented an initial impairment of progesterone production by modifying vasculature to provide ample substrate for hormone synthesis and increased the large luteal cell population to increase progesterone synthesis [15]. The aberrant morphology of leptin deficient lutea can be reversed when leptin replacement therapy is applied during the early stage of development [14]. However, unlike the leptin deficient CL, the early stage rescued CL exhibited accelerated development, appearing as a mature stage CL with increased tissue area and large luteal cell size (Table 2) [14,16-18]. Interestingly, both FGF2 and leptin were localized on the cell membrane and in the cytosol of large luteal cells of the rescued CL. This observation may explicate the greater size of the large luteal cells in the rescued CL in that FGF2 promotes both angiogenesis and the proliferation and differentiation of steroidogenically active luteal cells [19]. Collectively, the induced luteal leptin deficiency may have increased tissue sensitivity to leptin, which promoted compensatory development upon hormone replacement. In summary, leptin appears to contribute to the development of the CL by facilitating a normal vascular landscape, potentially through angiogenic growth promotants, that influence normal luteal morphological formation. Future investigation will explore the mechanism through which leptin influences luteal development and the potential impact on the maintenance of a gravid CL.

## Figures and Tables

**Table 1:** Microscopic morphology of mature CL from control and leptin antibody treatment groups.

Treatment	Avg # of large luteal cells per area*#	Avg. # of small luteal cells per area*#	Ratio of large:small luteal cells per area*#	Avg. large vessel diameter* (mm)
Control	59.20 ± 1.54^a^	43.94 ± 2.15^a^	1.4 ± 0.08^a^	21.3 ± 0.03^a^
Leptin Antibody	76.3 ± 1.79^b^	33.11 ± 1.16b	2.3 ± 0.32^b^	33.0 ± 0.33^b^

a,b Superscripts indicates means different between treatment groups (P<0.01);

* Effect of treatment is significant (P<0.001);

# Area of tissue=26.6 × 10^4^ μm^2^ at 20× magnification

⌘Published data [14] and adapted for commentary

**Table 2:** Microscopic morphology of developing CL from control and leptin antibody+leptin treatment group.

Treatment	Avg # of large luteal cells per area*#	Avg. # of small luteal cells per area*#	Ratio of large:small luteal cells per area*#	Avg. large luteal cell size per area*# (μm
Control	235.84 ± 6.11^a^	82.92 ± 3.25^a^	3.06 ± 0.08^a^	16.32 ± 0.65^a^
Leptin Antibody+Leptin	85.32 ± 3.34^b^	51.76 ± 2.43^b^	1.86 ± 0.06^b^	22.56 ± 0.70^b^

a,b Superscripts indicates means different between treatment groups (P<0.0001);

* Effect of treatment is significant (P<0.0001);

# Area of tissue=26.6 × 10^4^ μm^2^ at 20× magnification

⌘ Published data [14] and adapted for commentary

## References

[1] Reynolds LP, Grazul-Bilska AT, Killilea SD, Redmer DA (1994). Mitogenic factors of corpora lutea. Progress in Growth Factor Research.

[2] Redmer DA, Reynolds LP (1996). Angiogenesis in the ovary. Rev Reprod.

[3] Bopp B, Shoupe D (1993). Luteal phase defects. J Reprod Med.

[4] Balasch J, Vanrell JA (1987). Corpus luteum insuffciency and fertility: A matter of controversy. Hum Reprod.

[5] Coomarasamy A, Williams H, Truchanowicz E, Seed PT, Small R (2015). A randomized trial of progesterone in women with recurrent miscarriages. N Engl J Med.

[6] Susuki T, Sasano H, Takaya R, Fukaya T, Yajima A (1998). Cyclic changes of vasculature and vascular phenotypes in normal human ovaries. Hum Reprod.

[7] Reynolds LP, Redmer DA (1998). Expression of the angiogenic factors, basic fbroblast growth factor and vascular endothelial growth factor, in the ovary. Am Soc Anim Sci.

[8] Shalaby F, Rossant J, Yamaguchi TP, Gertsenstein M, Wu XF (1995). Failure of blood-island formation and vasculogenesis in Flk-1 defcient mice. Nature.

[9] Aleff S, Petrai I, Bertolani C, Parola M, Colombatto S (2005). Upregulation of proinfammatory and proangiogenic cytokines by leptin in human hepatic stellate cells. Hepatology.

[10] Cao R, Brakenhielm RE, Wahlestedt C, Thyberg J, Cao Y (2001). Leptin induces vascular permeability and synergistically stimulates angiogenesis with FGF-2 and VEGF. Proc Natl Acad Sci U S A.

[11] Spicer LJ, Francisco CC (1997). The adipose obese gene product, leptin: Evidence of a direct inhibitory role in ovarian function. Endocrinology.

[12] Spicer LJ, Francisco CC (1998). Adipose obese gene product, leptin, inhibits bovine ovarian thecal cell steroidogenesis. Biol Reprod.

[13] Wiles JR, Katchko RA, Aguirre EA, Mahan MD, O'Gorman CW (2014). The effect of leptin on luteal angiogenic factors during the luteal phase of the estrous cycle in goats. Anim Reprod Sci.

[14] Ramirez MA, Arellano AA, Xie F, Benavides EA, Katchko RA (2017). Role of leptin in the development of the corpus luteum. Leptin: Production, regulation and functions.

[15] Wiltbank MC, Niswender GD (1992). Functional aspects of differentiation and degeneration of the steroidogenic cells of the corpus luteum in domestic ruminants. Anim Reprod Sci.

[16] AZMI TI, Bongso TA (1985). The Ultrastructure of the corpus luteum of the goat. Pertanika.

[17] Kalender H, Arikan S (2007). Size distribution of dispersed luteal cells during oestrous cycle in Angora goats. Reprod Domest Anim.

[18] Wiltbank MC (1994). Cell types and hormonal mechanisms associated with mid-cycle corpus luteum function. J Anim Sci.

[19] Grazul-Bilska AT, Redmer DA, Jablonka-Shariff A, Biondini ME, Reynolds LP (1995). Proliferation and progesterone production of ovine luteal cells from several stage of the estrous cycle: effects of fbroblast growth factors and luteinizing hormone. Can J Physiol Pharmacol.

